# Competition and regional Phillips curve: Evidence from China

**DOI:** 10.1371/journal.pone.0301546

**Published:** 2024-05-16

**Authors:** Shukai Du

**Affiliations:** School of Finance, Shanghai University of Finance and Economics, Shanghai, China; University of Education, PAKISTAN

## Abstract

The product market competition affects the non-neutrality of monetary policy. This paper quantitatively assesses its impact on the slope of the Phillips curve through the channels of nominal and real rigidity. We build a New Keynesian model using the Kimball aggregator and the Calvo staggered pricing scheme. We show that a more competitive market environment has opposite effects on the slope of the Phillips curve by increasing the real rigidity and lowering the nominal rigidity. We then estimated the model using regional data of China. The Bayesian estimation shows that the response of inflation-output trade-off is larger in the region with a high degree of competition. Counterfactual experiments demonstrate that nominal rigidity has a dominant role and accounts for the majority of the difference in the Phillips curve, while the contribution of real rigidity is relatively minor. Our results highlight the key role of nominal rigidity in determining the inflation-output dynamics.

## 1. Introduction

The relationship between inflation and economic activity, summarized by the Phillips curve, is fundamental to monetary theory and policy analysis. In theory, this relationship is affected by the degree of competition. However, competition can have opposite impacts on the Phillips curve through the real and nominal rigidity. On the one hand, a higher degree of competition can make a firm’s elasticity of demand more sensitive to its relative price, thereby reducing the extent of price adjustments. This leads to a larger real rigidity and flattens the Phillips curve [1]. On the other hand, a more competitive environment makes firms pay more attention to their competitors, and adjust their prices more frequently [2, 3]. This lowers the nominal rigidity and steepens the Phillips curve. Due to modeling and data challenges, little quantitative results are known when both nominal and real rigidity are considered.

Existing empirical studies provide an ambiguous view on how competition affects the direction of price rigidity. Some studies have demonstrated a negative correlation between market concentration and price stickiness, suggesting that increased market competition leads to higher frequency of price adjustments by firms [4–7]. This implies that as market competition intensifies, the regional Phillips curve tends to flatten. However, other empirical research has produced contrary findings. Domburg [8] observed that in the British market, higher levels of market concentration were associated with faster price adjustments by firms. Setiawan et al. [9, 10] also found a positive relationship between industry concentration and price rigidity in Indonesia. The insufficient differentiation between nominal and real rigidity channels is one of the key reasons for the divergence in conclusions of existing empirical studies.

In this paper, we quantitatively evaluate the impact of real rigidity and nominal rigidity on the slope of Phillips curve by exploring regional difference in competition across China. There are two reasons for using regional data from China. First, effectively distinguishing demand and supply shocks is essential for identifying the slope of the Phillips Curve. Supply shocks generate positive correlations between inflation and output gaps, whereas demand shocks have the opposite effect. Several recent papers have point out that, compared to using national data, estimating the Phillips curve with regional data can better identify demand and supply shocks and improve the accuracy of the estimates [11–13].

**Fig 1:**
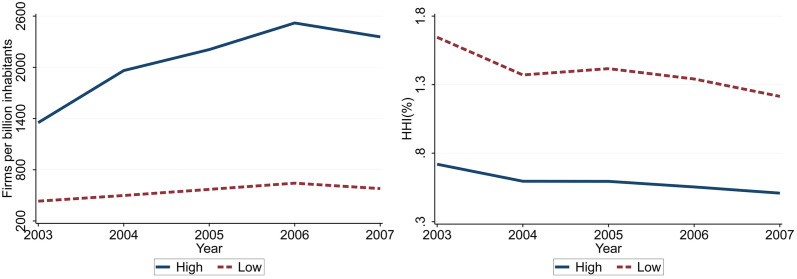
Competition in two regions. The left graph shows the average firm number per million inhabitants and the right graph displays the the sale weighted HHI for the two regions. The solid line denotes the high competition region and the dashed line denotes the low competition region. The high competition region consists the provinces with average firm number per billion inhabitants above the median. Our data are from ASIF which ends in 2007.

Second, the Chinese economy provides a natural setting for studying the effects of competition. The entry into WTO in 2001 [14] and the existence of market segmentation [15] have generated different degrees of competition across provinces in China. We use data from the Chinese Annual Survey of Industrial Firms (ASIF) to divide provinces into two groups according to the number of firms per inhabitants. The left graph in Fig 1 shows the average number of firms and the right graph computes the sale weighted Herfindahl-Hirschman Index (HHI) for each group. As shown in Fig 1, the high competition region has more firms and low level of market concentrations. It is interesting by itself to find out whether such large difference in competition generates large impact on the slope of Phillips curve.

To quantify the impacts of real rigidity and nominal rigidity, we build a structural New Keynesian model with the Kimball-style aggregator [16] and the Calvo price-setting. Unlike the Dixit-Stiglitz aggregator, the demand elasticity of each firm under the Kimball aggregator increases in its price relative to its competitors. Therefore, when a firm wants to raise prices, it will temper its price increase if some other firms cannot adjust prices. This strategic complementarity consists a form of real rigidity and is stronger with more firms.

We divide the economy into two regions with different degrees of market competitiveness. In this way, we can investigate the impact of competition on the Phillips curve by comparing the differences between regions. As the parameters in the Kimball aggregator are linked with the moments of firm number and markups, we calibrate these parameters on the regional level. We then estimate other parameters for nominal rigidity, money growth rule and shock processes with the Bayesian method for each region.

Our estimation shows that, there exists significant differences in both the nominal rigidity and real rigidity across regions in China. The posterior median of the Calvo pricing parameter, that is, the probability of not optimally setting the price, is 0.87 in the high competition region versus 0.94 in the low one. Therefore, the high competition region has larger real but low nominal rigidity. The ratio of inflation response to output response on impact is 0.14 in the high competition region and 0.06 in the low competition region. which means a flatter regional Phillips curve.

The previous result is the total impact of the real and nominal rigidity, but it cannot tell us how the slope difference is accounted by the real and nominal rigidity across regions. To answer this questions, we compute the response of inflation and output to the regional monetary supply shock and conduct counterfactual experiments by changing the firm numbers and the Calvo parameter of each region into its counterpart. The ratio of output to inflation for the high competition region increases to 0.15 from 0.14 when the firm number is reduced to the value of the low competition region, while it decreases to 0.05 when the Calvo parameter decreases to the low competition region value. When both real and nominal rigidity parameters are changed into the values of low competition region, the ratio changes to 0.06. Consequently, the nominal rigidity is more important in determining the slope of Phillips curve.

Our paper contributes to the literature on how competition changes the inflation-output trade-off in China. There is a growing study about the relationship between market concentration and the slope of the Phillips curve. Mongey [17] and Wang and Werning [18] emphasize that the market competition can affect the effectiveness of monetary policy through the real rigidity channel. Fujiwara and Matsuyama [19] provides a theoretical framework that demonstrates that increased market concentration leads to a flattening of the Phillips curve. Hoynck et al. [20] point out that there is a positive relationship between market concentration and price rigidity and provide empirical evidence based on U.S. data. However, there are few empirical studies on the effect of competition on the Phillips curve in developing countries, like China. It has been well-documented that that the competition structure in China has changed after it enters WTO [14, 21, 22]. However, its impact on the inflation-output trade-off for China is not well studied. Our paper fills up this gap with quantitative DSGE analysis.

Our paper is complementary to the literature on how competition from globalization changes the domestic inflation-output trade-off. Early research Sbordone [23] finds that the real rigidity from the competition is not large enough to flatten the Phillips curve in the U.S., but calibration from Watson [24] indicates the effect depends on the degree of openness non-linearly. Guerrieri et al. [25] finds that international competition lowers the traded goods prices sensitivity to output. These papers model the increase in competition as more foreign products in domestic markets. We instead model competition as the increase in the number of domestic firms, which is consistent with the above trade literature and our empirical evidence in Fig 1.

Last, our paper belongs to the literature on the flattening Phillips curve. Beside change in competition, other factors are proposed to explain the flatter Phillips curve in the U.S. after the financial crisis. They include globalization [26–28], the anchored expectations [29] and financial frictions [30, 31].

The remainder of this paper is organized as follows. In Section 2, we lay out the DSGE model with Kimball preference and nominal rigidity. We describe our data in Section 3. The Bayesian estimation of the DSGE model and the counterfactual experiments are present in Section 4. Section 5 concludes.

## 2. Model

We augment the standard New Keynesian model with the Kimball-type aggregator to incorporate the real rigidity from competition.

### 2.1 Household

The economy consists of two regions, indexed by *j* ∈ {*H*, *L*}. Each region has a representative, infinitely-lived household whose utility function, depends on the streams of period utility from consumption, *C*_*j*,*t*_, labor supply, *L*_*j*,*t*_, and real cash balance holdings, *M*_*j*,*t*_/*P*_*j*,*t*_:

$$\begin{array}{c} U_{j,t}=E_{t}\sum_{k=0}^{\infty }\beta^{k}[\frac{C_{j,t+k}^{1-\sigma }}{1-\sigma }-\frac{L_{j,t+k}^{1+\varphi }}{1+\varphi }+\frac{{\left(M_{j,t+k}/P_{j,t+k}\right)}^{1-\nu }}{1-\nu }], \end{array}$$ (1)

where *β*, *σ*, *φ*, and *ν* are the intertemporal discount factor, the inverses of consumption, labor supply, and real cash balance elasticities, respectively.

The household budget constraints is

$$\begin{array}{c} P_{j,t}C_{j,t}+B_{j,t}+M_{j,t}=W_{j,t}L_{j,t}+\left(1+i_{j,t-1}\right)B_{j,t-1}+M_{j,t-1}, \end{array}$$ (2)

where *P*_*j*,*t*_ is the price index, *B*_*j*,*t*_ denotes the holding of a nominal risk-free bond, *W*_*j*,*t*_ is the nominal wage, and *i*_*j*,*t*_ is the nominal risk-free interest rate. We assume that the bond markets are segmented from each other. As a result, the interest rates in the different regions are determined by the local supply and demand of bonds. In addition, a non-Ponzi condition on household real bond borrowing is imposed.

The first order conditions derived from the household problem are as follows:

$$\begin{array}{c} \frac{1}{C_{j,t}^{\sigma }}=\beta E_{t}\left[\frac{\left(1+i_{j,t}\right)P_{j,t}}{C_{j,t+1}^{\sigma }P_{j,t+1}}\right] \end{array}$$ (3)

$$\begin{array}{c} L_{j,t}^{\varphi }=\frac{W_{j,t}}{C_{j,t}^{\sigma }P_{j,t}} \end{array}$$ (4)

$$\begin{array}{c} {\left(\frac{M_{j,t}}{P_{j,t}}\right)}^{-\nu }=C_{j,t}^{-\sigma }\left[1-\beta \frac{C_{j,t}^{\sigma }}{C_{j,t+1}^{\sigma }}\frac{P_{j,t}}{P_{j,t+1}}\right] \end{array}$$ (5)

After the log-linearization, we obtain the Euler equation, labour supply and money demand functions for the household sector:

$$\begin{array}{c} {\hat{c}}_{j,t}=E_{t}{\hat{c}}_{j,t+1}-\frac{1}{\sigma }E_{t}\left({\hat{i}}_{j,t}-\pi_{j,t+1}\right) \end{array}$$ (6)

$$\begin{array}{c} \varphi {\hat{l}}_{j,t}={\hat{rw}}_{j,t}-\sigma {\hat{c}}_{j,t} \end{array}$$ (7)

$$\begin{array}{c} {\hat{gm}}_{j,t}=\pi_{j,t}+{\hat{c}}_{j,t}-{\hat{c}}_{j,t-1}-\frac{1}{\nu (1+\bar{i})}\left(i_{j,t}-i_{j,t-1}\right) \end{array}$$ (8)

where, ${\hat{rw}}_{j,t}=log\left(W_{j,t}\right)/P_{j,t}-log\left(W_{j}\right)/P_{j}$ is the log deviation of real wage in region *j*. And ${\hat{gm}}_{j,t}={\hat{m}}_{j,t}-{\hat{m}}_{j,t-1}$ is the log deviation of the growth rate of the nominal money demand.

The final consumption good, *C*_*j*,*t*_, is produced by perfectly competitive firms, which combine a continuum of intermediate goods, *C*_*j*,*i*,*t*_, according to:

$$\begin{array}{c} \int_{0}^{N_{j}}f\left(\frac{C_{j,i,t}}{C_{j,t}}\right)di=1, \end{array}$$ (9)

where *N*_*j*_ is the number of firm in region *j*, reflecting the degree of competition, $\frac{C_{j,i,t}}{C_{j,t}}$ is the market share of commodity produced by firm *i* and *f*(⋅) is an increasing, strict concave function. Following Dotsey and King [32], we choose the functional form of *f*(⋅) as:

$$\begin{array}{c} f\left(\frac{C_{j,i,t}}{C_{j,t}}\right)=\frac{1}{\left(1+\eta \right)\gamma_{j,t}}{\left[\left(1+\eta \right)\frac{C_{j,i,t}}{C_{j,t}}-\eta \right]}^{\gamma_{j,t}}-\frac{1}{N_{j}}[\frac{1}{\left(1+\eta \right)\gamma_{j,t}}-1] \end{array}$$ (10)

Following Guerrieri et al. [25], we assume that the elasticity of substitution, *γ*_*j*,*t*_ > 0, is time-varying to introduce the cost-push shocks in the Phillips curve. Specifically, we set cost-push shock $\psi_{j,t}=-\left(log\left(\gamma_{j,t}/\bar{\gamma }\right)\right)$, where $\bar{\gamma }$ is the steady state value and *ψ*_*j*,*t*_ follows an AR(1) process: $\psi_{j,t}=\rho_{\psi }\psi_{j,t-1}+\varepsilon_{j,t}^{\psi }$, where *ρ*_*j*,*ψ*_ ∈ [0, 1) and $\varepsilon_{j,t}^{\psi }∼N\left(0,\sigma_{j,\psi }^{2}\right).$ The parameter *η* < 0 controls the curvature of the demand curve and introduces the variable elasticity demand for firms. When *η* = 0 and $\gamma_{j,t}=\bar{\gamma }$, the aggregator function reduces to the one with constant elasticity of substitution.

### 2.2 Firms

There is a continuum of monopolistic competitive firms in the economy. Each firm produces one product and is endowed with a decreasing return to scale production technology:

$$\begin{array}{c} Y_{j,i,t}=A_{j,t}L_{j,i,t}^{1-a}, \end{array}$$ (11)

where *Y*_*j*,*i*,*t*_ is the output for firm i at time t and *L*_*j*,*i*,*t*_ is the input of labor in production. The log of regional total factor productivity follows an *AR*(1) process $lnA_{j,t}=\rho_{j,a}lnA_{j,t-1}+\varepsilon_{j,t}^{a}$, *ρ*_*j*,*a*_ ∈ [0, 1) and $\varepsilon_{j,t}^{a}∼N\left(0,\sigma_{j,a}^{2}\right)$. Different from the standard setting, the measure of firms is *N*_*j*_ in region *j*, instead of 1.

Under the Kimball aggregator, the market share of firm *i* at time *t*, *x*_*j*,*i*,*t*_, is

$$\begin{array}{c} x_{j,i,t}=\frac{C_{j,i,t}}{C_{j,t}}=\frac{1}{\left(1+\eta \right)}{\left(\frac{P_{j,i,t}}{P_{j,t}^{c}}\right)}^{\frac{1}{\gamma_{j,t}-1}}+\frac{\eta }{1+\eta }, \end{array}$$ (12)

where *P*_*j*,*i*,*t*_ is the price of individual product *P*_*j*,*t*_ is the price index and $P_{j,t}^{c}$ is the competition-based aggregate price index,

$$\begin{array}{c} P_{j,t}^{c}={\left[\int_{0}^{N_{j}}{\left(P_{j,i,t}\right)}^{\frac{\gamma_{j,t}}{\gamma_{j,t}-1}}di\right]}^{\frac{\gamma_{j,t}-1}{\gamma_{j,t}}}. \end{array}$$ (13)

The consumption-based aggregate price index in the consumer budget constraint, *P*_*i*,*t*_, is defined by,

$$\begin{array}{c} P_{j,t}=\frac{1}{1+\eta }{\left[\int_{0}^{N_{j}}{\left(P_{j,i,t}\right)}^{\frac{\gamma_{j,t}}{\gamma_{j,t}-1}}di\right]}^{\frac{\gamma_{j,t}-1}{\gamma_{t}}}+\frac{\eta }{1+\eta }\int_{0}^{N_{j}}P_{j,i,t}di. \end{array}$$ (14)

Under Kimball aggregator, the price elasticity of demand *θ*_*j*,*i*,*t*_ is no longer a constant but an increasing function of a firm’s relative price:

$$\begin{array}{c} \theta_{j,i,t}=-\frac{1}{\left(\gamma_{j,t}-1\right)}{\left[1+\eta {\left(\frac{P_{j,i,t}}{P_{j,t}}\right)}^{\frac{1}{1-\gamma_{j,t}}}{\left(\frac{P_{j,t}}{P_{j,t}^{c}}\right)}^{\frac{1}{1-\gamma_{j,t}}}\right]}^{-1}. \end{array}$$ (15)

The demand elasticity increases as the market becomes more competitive. The value of super-elasticity, the elasticity of demand elasticity concerning a firm’s relative price, also becomes larger. When there is an increase in the aggregate demand, had a firm decided to re-set its price, it would curtail its price increase and set a smaller markup over its marginal cost. Thus, the market competition increases the real rigidity.

### 2.3 The New Keynesian Phillips curve

Firms adjust their prices following the Calvo-style pricing contracts. In each period, firms can re-optimize the price with a probability of (1 − *ξ*_*j*_). The probability is independent across time and firms. In each period, firms that cannot re-optimize their prices reset their prices according to lagged inflation as in Christiano et al. [33]. In particular,

$$\begin{array}{c} P_{j,i,t}={\left(1+\pi \right)}^{1-\delta_{j,d}}{\left(1+\pi_{j,t-1}\right)}^{\delta_{j,d}}P_{j,i,t-1}, \end{array}$$ (16)

where *π*_*j*,*t*−1_ = *log*(*P*_*j*,*t*−1_) − *log*(*P*_*j*,*t*−2_) is the inflation rate and *π*_*j*_ is the steady state of *π*_*j*,*t*_. The parameter *δ*_*j*,*D*_ ∈ [0, 1] is the degree of indexation. If *δ*_*j*,*D*_ = 0 and *π*_*j*_ = 0, the price setting reduces to standard one without indexation.

When firm *i* can re-optimize its price in period *t*, it maximizes the expected discounted value of profit,

$$\begin{array}{c} E_{t}\sum_{k=0}^{\infty }\{\xi_{j}^{k}[Q_{j,t+k}I_{j,t,t+k}P_{j,i,t}Y_{j,t+k}x_{j,i,t}\left(\frac{I_{j,t,t+k}P_{j,i,t}}{P_{j,t+k}^{c}}\right)\\ -C(Y_{j,t+k}x_{j,i,t}\left(\frac{I_{j,t,t+k}P_{j,i,t}}{P_{j,t+k}^{c}}\right))]\} \end{array}$$ (17)

taking the process of stochastic discount factor of household *Q*_*j*,*t*+*k*_, the household demand *x*_*j*,*i*,*t*_, the aggregate prices, the wage rate, and price indexation *I*_*j*,*t*,*t*+*k*_ as given. In the equation, *C*(⋅) is the firm’s cost function.

The first-order condition for the firm’s optimal price is

$$\begin{array}{cc} 0= & \frac{1}{P_{j,i,t}^{o}}E_{t}\sum_{k=0}^{\infty }\{\xi_{j}^{k}Q_{j,t+k}Y_{j,t+k}x_{j,i,t}\left(\frac{I_{j,t,t+k}P_{j,i,t}^{o}}{P_{j,t+k}^{c}}\right)\left(\theta_{j,i}\left(x_{j,i,t}\left(\frac{I_{j,t,t+k}P_{j,i,t}^{o}}{P_{j,t+k}^{c}}\right)\right)-1\right)\\ & [\frac{I_{j,t,t+k}P_{j,i,t}^{o}}{P_{j,t+k}}-\mu_{j,i}\left(x_{j,i,t}\left(\frac{I_{j,t,t+k}P_{j,i,t}^{o}}{P_{j,t+k}^{c}}\right)\right)MCR_{j,i,t}\left(Y_{j,t+k}x_{j,i,t}\left(\frac{I_{j,t,t+k}P_{j,i,t}^{o}}{P_{j,t+k}^{c}}\right)\right)]\}, \end{array}$$ (18)

where $P_{j,i,t}^{o}$ is the optimal price chosen by firm *i* at time t in region *j*, and *MCR*_*j*,*i*,*t*_ is the real marginal cost. After the log-linearizing Eq 18 around a zero inflation steady state and use the fact that log(*P*_*t*_) equals $\text{log}(P_{t}^{c})$ at first order approximation, we have the New Keynesian Phillips curve,

$$\begin{array}{c} \pi_{j,t}=\frac{\delta_{d}}{1+\delta_{d}\beta }\pi_{j,t-1}+\frac{\beta }{1+\delta_{d}\beta }E_{t}\pi_{j,t+1}+\Theta_{j}{\tilde{y}}_{j,t}+k_{j}{\hat{\psi }}_{j,t} \end{array}$$ (19)

where ${\tilde{y}}_{j,t}={\hat{y}}_{j,t}-{\hat{y}}_{j,t}^{n}$ is the log-deviation of output from its natural level.

We express the slope coefficient of NKPC as Θ_*j*_ = Θ_*j*,*NR*_ × Θ_*j*,*RR*_, where Θ_*j*,*NR*_ and Θ_*j*,*RR*_ are positively correlated to the measurements of nominal rigidity and real rigidity respectively. The specific expressions of the coefficients are as follows:

$$\begin{array}{c} \Theta_{j,NR}=\frac{\left(1-\xi_{j}\beta \right)\left(1-\xi_{j}\right)}{\xi_{j}\left(1+\delta_{d}\beta \right)} \end{array}$$ (20)

$$\begin{array}{c} \Theta_{j,RR}=\frac{\sigma +\frac{\varphi +\alpha }{1-\alpha }}{1+{\bar{\theta }}_{j}\left(e_{j,\mu }^{x}+e_{j,mc}^{y}\right)}. \end{array}$$ (21)

In the expression, ${\bar{\theta }}_{j}$ is the price elasticity of demand at steady state. And the constants $e_{j,\mu }^{x}$ and $e_{j,mc}^{y}$, are the elasticity of markup to the firm’s market share and the elasticity of marginal cost to the firm’s output at steady state.

$$\begin{array}{c} {\bar{\theta }}_{j}=\frac{1}{\left(1-\bar{\gamma }\right)\left(1+\eta N_{j}^{\frac{1}{\bar{\gamma }}}\right)} \end{array}$$ (22)

$$\begin{array}{c} e_{j,\mu }^{x}=\frac{1}{1-{\bar{\theta }}_{j}}\eta N_{j}^{\frac{1}{\bar{\gamma }}} \end{array}$$ (23)

$$\begin{array}{c} e_{j,mc}^{y}=\frac{\alpha }{1-\alpha } \end{array}$$ (24)

Importantly, both nominal rigidity and real rigidity affect the response of inflation to the output. Θ_*j*_ can be decomposed into two parts. The first part, Θ_*j*,*NR*_, depends inversely on the Calvo pricing parameter, *ξ*_*j*_, so it increases as nominal rigidity decrease, and the Phillips curve becomes steeper. The second part, Θ_*j*,*RR*_, captures the impact of real rigidity. Since *η* < 0 and $\bar{\gamma }<1$, ${\bar{\theta }}_{j}$ rises as *N*_*j*_ grows. ${\bar{\theta }}_{j}\left(e_{j,\mu }^{x}+e_{j,mc}^{y}\right)$ contained in Θ_*j*,*RR*_ increases as *N*_*j*_ goes up. When *N*_*j*_ increase, the real rigidity increase, the coefficient Θ_*j*_ decreases and the Phillips curve becomes flatter.

The expression for the coefficient *k*_*j*_ before the cost shock ${\hat{\psi }}_{j,t}$ is as follows:

$$\begin{array}{c} k_{j}=\frac{\left(1-\xi_{j}\beta \right)\left(1-\xi_{j}\right)}{\xi_{j}\left(1+\delta_{d}\beta \right)}\frac{e_{j,\mu }^{\gamma }-e_{j,mc}^{\gamma }}{1+{\bar{\theta }}_{j}\left(e_{j,\mu }^{x}+e_{j,mc}^{y}\right)} \end{array}$$ (25)

where $e_{j,\mu }^{\gamma }$ and $e_{j,mc}^{\gamma }$ are the elasticity of the markup to *γ*_*j*,*t*_ and the elasticity of the marginal cost to *γ*_*j*,*t*_ at steady state.

$$\begin{array}{c} e_{j,\mu }^{\gamma }=\left(\frac{1}{{\bar{\theta }}_{j}-1}\right)\frac{\bar{\gamma }}{{\left(\bar{\gamma }-1\right)}^{2}}-{\bar{\theta }}_{j}\frac{1}{\bar{\gamma }-1}log\left(N_{j}\right)e_{j,\mu }^{x} \end{array}$$ (26)

$$\begin{array}{c} e_{j,mc}^{\gamma }={\bar{\theta }}_{j}\frac{1}{\bar{\gamma }-1}\frac{\alpha }{1-\alpha }log\left(N_{j}\right) \end{array}$$ (27)

### 2.4 Monetary supply

The monetary policy in China is mainly based on quantity control during our data sample from 2009 Q1 to 2019 Q4. Therefore, we follow Chen et al. [34] and Chang et al. [35] to use a money growth rule. We further allow the central bank to carry out its policy on the regional level. Specifically, the central bank adjusts the growth rate of the local money supply in response to local inflation gap and local output growth rate gap:

$$\begin{array}{c} {\hat{gm}}_{j,t}=\phi_{j,m}{\hat{gm}}_{j,t-1}+\left(1-\phi_{j,m}\right)\left(\phi_{j,\pi }\pi_{j,t}+\phi_{j,y}{\hat{gy}}_{j,t}\right)+\varepsilon_{j,t}^{m}, \end{array}$$ (28)

where ${\hat{gm}}_{j,t}$ and ${\hat{gy}}_{j,t}$ are the log deviations of growth rate of monetary supply and output in region *j*. The coefficient *ϕ*_*j*,*m*_ ∈ [0, 1) measures the smoothness of monetary policy, and the coefficient *ϕ*_*j*,*π*_ and *ϕ*_*j*,*y*_ measure the response strength to the deviation. The random term ${\varepsilon_{j,t}}^{m}∼N\left(0,{\sigma_{j,m}}^{2}\right)$ is the regional monetary policy shock.

At the national level, the money supply is the sum of regional ones, *M*_*t*_ = *M*_*H*,*t*_ + *M*_*L*,*t*_. In terms of growth rate approximated around the steady state, the national monetary growth rate *gm*_*t*_ is the weighted sum of regional ones:

$$\begin{array}{c} {\hat{gm}}_{t}=\omega {\hat{gm}}_{H,t}+\left(1-\omega \right){\hat{gm}}_{L,t} \end{array}$$ (29)

where $\omega =\frac{M_{H}}{M_{H}+M_{L}}$ is the proportion of nominal money holdings by the household in region *H* at steady state.

See S1 Appendix for derivation of the New Keynesian Phillips curve and model equilibrium conditions.

## 3. Data

Our data contains quarterly time series of provincial GDP, provincial CPI, and national monetary indicators from China. We collect the series of province-level gross regional product from the National Bureau of Statistics of China (NBSC) and the province-level month-on-month CPI from the WIND database as the measurement for inflation. We don’t use the GDP deflator or PPI because the former is only available at the national level, and the latter starts from 2015. The sample period starts from 2009Q1 to 2019Q4 to avoid the large shock of 2007 financial crisis and 2019 Covid pandemic, which lead to structural changes in the economy and makes our estimation unstable.

To examine how market competition affects regional Phillips curves, we divide the provinces in mainland China into two groups, the high competition region (*H*) and the low competition region (*L*), according to the number of firms per inhabitants at the province level. Data on the number of provincial enterprises are derived from China’s Annual Survey of Above-scale Industrial Firms (ASIF), and we obtain information on population from the NBSC. Each group consists 15 provinces.

We construct the real GDP and CPI series for the high and low competition region, respectively. The regional real GDP is the total sum of provinces in the group, and the regional CPI is weighted by provincial real GDP. We also construct the real GDP and CPI series for the nation from the regional data. The monetary policy mainly adopts quantity control during our sample period in China, we collect data on money supply from the Wind database. In our baseline estimation, we use the log difference of M2 as a measurement for money growth rate. The results remain robust when using aggregate financing as a measurement for the money supply.

In sum, there are five observable quarterly detrended time series used in our Bayesian SVAR estimation, {*gm*_*t*_, *gy*_*H*,*t*_, *gy*_*L*,*t*_, *π*_*H*,*t*_, *π*_*L*,*t*_}. They are the national money supply growth rate, the output growth rates and inflation rates of *H* and *L* regions. For all the data in the estimation, we seasonally adjusted the series using the X-13 method and then remove the local mean via a bi-weight filter following Stock and Watson [36]. We report the data series used for Bayesian estimation in S1 Dataset. For more information of the data, see S1 Appendix.

## 4. Bayesian DSGE estimation

In this section, we first calibrate part of parameters in the model, then we estimate the log-linearized model using Bayesian methods. After the estimation, we conduct counterfactual experiments on the nominal and real rigidity parameters to investigate their relative importance in determining the inflation and output dynamics.

### 4.1 Calibrated parameters

We set a subset of parameters constant before the Bayesian estimation. The discount factor is set to *β*=0.995, which is consistent with Chang et al. [35]. The CRRA parameter *σ*, the inverse of the Frisch elasticity of labor supply *φ*, and the interest rate elasticity of money demand *ν* are set to 2. The labor share *α* is set to 0.5 according to Zhu [37] and Chang et al. [35].

Parameters *η* and $\bar{\gamma }$ in the Kimball aggregator are important in determining the curvature of the demand function and the real rigidity in the model. We choose *η* and $\bar{\gamma }$ to match the firm number *N* and average markup in both H and L regions in 2007. The firm numbers for both regions are computed from Chinese Annual Survey of Industrial Firms (ASIF). By normalizing *N*_*L*_ = 1, we have *N*_*H*_ = 4.067.

The regional markup is the average of the local firm-level markups weighted by sales. We estimate the firm-level markups using the method in De Loecker and Warzynski [38] and Brandt et al. [14]. These methods control the various inputs in estimating the firm-level markups. The data used to calculate the markup are also from the ASIF. The average markup in the high competition region is *μ*_*H*_ = 1.198, lower than its counterpart in the low competition region *μ*_*L*_ = 1.235. Given the firm numbers and the markups, we compute *η* = −0.028 and $\bar{\gamma }=0.804$ by solving the following two equations:

$$\begin{array}{c} \frac{1}{\bar{\mu_{H}}}=1+\left(\bar{\gamma }-1\right)\left(1+\eta {N_{H}}^{\frac{1}{\bar{\gamma }}}\right) \end{array}$$ (30)

$$\begin{array}{c} \frac{1}{\bar{\mu_{L}}}=1+\left(\bar{\gamma }-1\right)\left(1+\eta {N_{L}}^{\frac{1}{\bar{\gamma }}}\right), \end{array}$$ (31)

which are the steady state equations for the markup and the number of firms.

We observe the national level of money supply, but the regional level of money supply is not observable, so we use yearly average regional loan data to approximate the money holdings share of *H* region *ω*. According to our calculation based on data from 2009 to 2019, the average value of *ω* during the sample period is 0.672.

As Eichenbaum and Fisher [39] discussed, one cannot separately identify the nominal and real rigidity using aggregate data. We face the same challenge when we want to identify them for each region. Following the literature, we first calibrate the real-rigidity parameters *η* and $\bar{\gamma }$ with markup computed from micro-data, then estimate the nominal-rigidity parameter with the Bayesian method. Table 1 summarizes all calibrated parameters.

**Table 1:** Calibrated parameters.

Parameter	Description	Value
A.Household		
*σ*	Inverse of the elasticity of intertemporal substitution	2
*φ*	Inverse of the Frisch elasticity	2
*ν*	Demand for real balance	2
*β*	Discount factor	0.995
*α*	Output elasticity of labor	0.5
*η*	Shape parameter of demand function	-0.028
$\bar{\gamma }$	Shape parameter of demand function	0.804
B.Firms		
*N*_*H*_	Relative firm number in *H* region	4.067
*N*_*L*_	Normalized firm number in *L* region	1
C.Monetary policy		
*ω*	the money holdings share of *H* region	0.672

### 4.2 Estimated results

We adopt the Calvo assumption, so our model has no direct built-in response of nominal rigidity to the degree of market competition. To capture the impact of competition on nominal rigidity we estimate the Calvo parameter based on data from different regions. The variations in data will tell us how nominal rigidity varies with market competition.

Table 2 summarizes the distribution type, the mean and standard deviation of the priors on the left. We harmonize the priors on the estimated parameters for both regions as much as possible. The Calvo price stickiness parameters *ξ*_*j*_ are Beta distributed with mean 0.5 and standard deviation 0.1. The degree of indexation *δ*_*d*_ is also Beta distributed. In addition, we assume that *δ*_*d*_ is same across regions in the baseline estimation. This assumption helps us to focus on the difference brought from the real rigidity and nominal rigidity directly. We will relax this restriction in our robust exercise. The persistence of the monetary policy rule and the technology shocks cost shocks are set as Beta distributed with different means. The response coefficients in the monetary policy rule are set as Normal distributed with negative means and 0.1 standard deviations. The standard deviations of shocks are assumed to follow inverse Gamma distributions with a mean of 5 percent and a 0.1 variance. The right panel of Table 2 reports the mean, the median, and the 5 and 95 percentiles of the posterior distribution of the parameters obtained by the Metropolis-Hastings algorithm. We also report the prior and post distributions in S1 Appendix.

**Table 2:** Bayesian estimation results.

	Prior distribution			Posterior distribution			
	Distribution	Mean	Std.Dev.	Mean	Median	5%	95%
A. Nominal price ridigity							
*ξ*_*H*_	Beta	0.500	0.100	0.866	0.870	0.821	0.914
*ξ*_*L*_	Beta	0.500	0.100	0.938	0.942	0.924	0.953
*δ*_*d*_	Beta	0.400	0.100	0.312	0.308	0.185	0.426
B. Monetary policy							
*ϕ*_*H*,*π*_	Normal	-1.000	0.100	-1.197	-1.194	-1.332	-1.067
*ϕ*_*H*,*y*_	Normal	-0.300	0.100	-0.375	-0.375	-0.512	-0.247
*ϕ*_*H*,*m*_	Beta	0.400	0.100	0.446	0.447	0.353	0.546
*σ*_*H*,*m*_	Inv.Gamma	0.050	0.100	0.022	0.021	0.018	0.025
*ϕ*_*L*,*π*_	Normal	-1.000	0.100	-0.884	-0.884	-1.037	-0.733
*ϕ*_*L*,*y*_	Normal	-0.300	0.100	-0.386	-0.386	-0.537	-0.251
*ϕ*_*L*,*m*_	Beta	0.400	0.100	0.562	0.566	0.416	0.722
*σ*_*L*,*m*_	Inv.Gamma	0.050	0.100	0.051	0.050	0.040	0.061
C. Shocks							
*ρ*_*H*,*a*_	Beta	0.700	0.100	0.972	0.974	0.964	0.984
*ρ*_*H*,*ψ*_	Beta	0.500	0.100	0.303	0.300	0.205	0.403
*σ*_*H*,*a*_	Inv.Gamma	0.050	0.100	0.115	0.112	0.082	0.143
*σ*_*H*,*ψ*_	Inv.Gamma	0.050	0.100	0.206	0.192	0.095	0.292
*ρ*_*L*,*a*_	Beta	0.700	0.100	0.636	0.631	0.429	0.870
*ρ*_*L*,*ψ*_	Beta	0.500	0.100	0.494	0.492	0.350	0.641
*σ*_*L*,*a*_	Inv.Gamma	0.050	0.100	0.357	0.355	0.118	0.532
*σ*_*L*,*ψ*_	Inv.Gamma	0.050	0.100	0.046	0.036	0.013	0.073

The posterior distribution is constructed by the Metropolis-Hastings algorithm with a single chain of 100000 draws, after dropping 100000 draws as a burn-in.

From the structural estimation results, nominal price rigidity, monetary growth rate and local shock processes are different between regions. In particular, the high competition region has a smaller mean of *ξ* than its counterpart in the low competition region. There are more firms optimally choosing their prices each period in the high competition region. For the regional monetary growth rates, both response negatively to the detrended regional GDP growth rate and inflation rate, which is consistent with Chen et al. [34]. The monetary growth rate in high competition region reacts more to inflation but less persistent. In addition, the high competition region has a smaller and more persistent TFP shocks, but a smaller and less persistent desired markup shock. All the differences in the estimations indicate different regional dynamics over business cycles.

Because of the existence of price indexation, when calculating the price adjustment duration, we count both firms that optimally change their prices and those passively adjust the prices based on last period inflation. Thus, the average price duration is nearly 2.5 quarters in the high competition region and 2.8 quarters in the low competition region, which is slightly longer than the average of 6.7 months for US [40].

**Fig 2:**
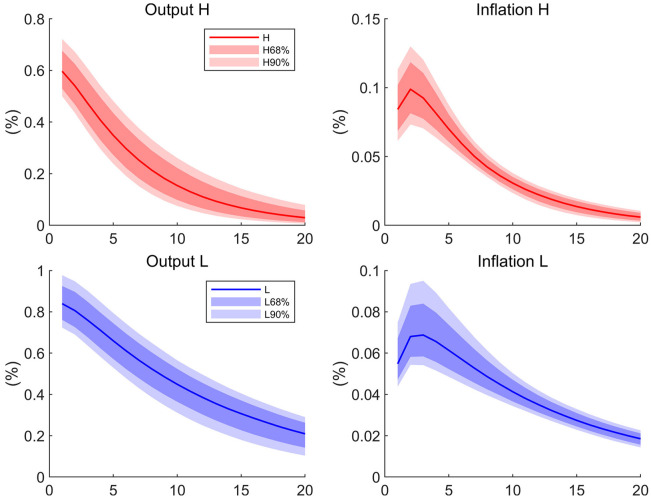
Impulse responses to monetary policy shocks. The four graphs depict the impulse responses of output and inflation to a positive one percent monetary supply shock. The solid lines are the median of the posterior distribution and the shaded areas are the corresponding confidence interval. *H* denotes the high-competition region and *L* the low-competition region.

We are interested in the response of output and inflation to the monetary policy shock across regions. The impulsive responses of output and inflation to an unexpected regional monetary growth shock are shown in Fig 2. After a positive monetary growth shock of 1 percent in high competition region, the output increased 60.7 bps and gradually returns to its steady state, meanwhile the inflation jumps to 8.4 bps, rises to 9.9 bps at peak and slowly returns to its steady state. In contrast, 1 percent positive monetary growth shock in the low competition region raises the output more (84.0 bps) and inflation (5.5 bps) less on impact.

To demonstrate the dynamics of the inflation-output trade-off over time, we compute the output Phillips multiplier in the lines of Barnichon and Mesters [41]. For a given exogenous shock, the multiplier is the ratio of the average reactions of inflation to output over time.

$$\begin{array}{c} M_{t}={\bar{R}}_{t}^{\pi }/{\bar{R}}_{t}^{y},\;\text{for}\;t=0,1,2,\ldots , \end{array}$$ (32)

where ${\bar{R}}_{t}^{\pi }=\frac{1}{t}\Sigma_{i=0}^{t}R_{i}^{\pi }$ and ${\bar{R}}_{t}^{y}=\frac{1}{t}\Sigma_{i=0}^{t}R_{i}^{y}$ are the average impulse responses of inflation and output at period *t*.

**Fig 3:**
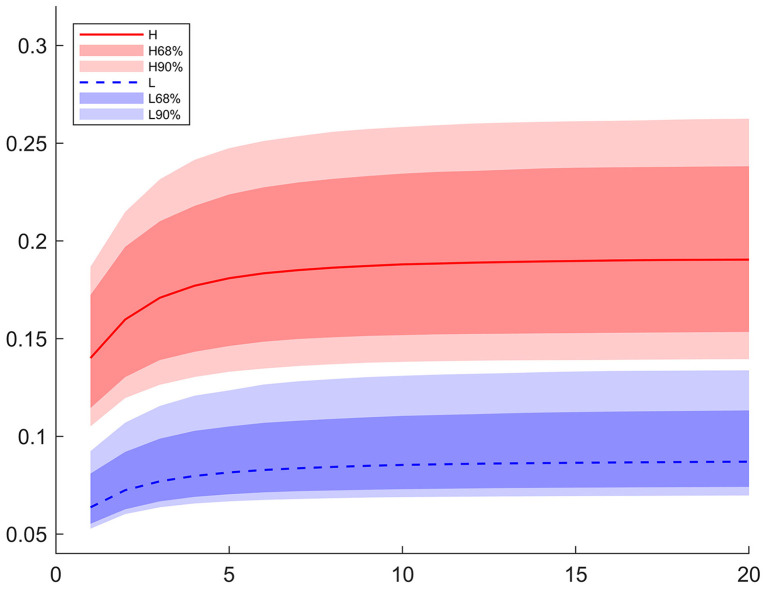
Phillips multipliers to monetary policy shocks. The graph depicts the Phillips multipliers to a positive one percent monetary policy shocks. The ratio immediately after the monetary policy shock is the slope of the Phillips curve. The solid lines are the median of the posterior distribution and the shaded areas are the corresponding confidence interval. *H* denotes the high-competition region and *L* the low-competition region.

In our case, we choose the regional monetary growth shock as the exogenous shock. The Fig 3 displays the regional Phillips multiplier over time computed from the posterior distributions. Importantly, the ratio of inflation response to output response on impact captures the inflation-output trade-off along the aggregate supply curve immediately after the monetary growth rate shock, which is the slope of the Phillips curve. The ratio in the high competition region is almost 2.2 times larger than the one in the low competition region. As competition increases real rigidity, it reduces nominal rigidity. This result suggests that the nominal rigidity dominates the real rigidity in determining the slope of the Phillips curve. Therefore, our estimation shows that the Phillips curve is much flatter in the low competition region.

### 4.3 Counterfactual experiments

The previous responses of high and low competition regions to the monetary shock are generated from different combinations of nominal and real rigidity. It cannot tell us how sensitive the inflation-output trade-off is to each type of rigidity, or how important each type of rigidity is across regions. To separate their impacts, we conduct the counterfactual experiments by changing the real and nominal rigidity of each region, respectively.

**Fig 4:**
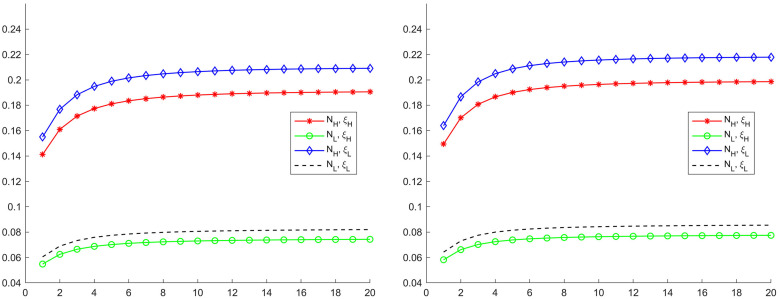
Phillips multipliers in counterfactual experiments. Each graph displays four cases of Phillips multipliers after a positive one percent money growth rate shock. The ratio immediately after the monetary policy shock is the slope of the Phillips curve. The line labeled with (*N*_*j*_, *ξ*_*j*_) denotes the Phillips multipliers computed under estimates from the high-competition (*j* = *H*) or the low-competition region (*j* = *L*). The left graph reports results for the high competition region and the right graph reports results for the low competition region.

Starting from the high competition region, we change the firm number *N*_*H*_ and the Calvo pricing parameter *ξ*_*H*_ into their low competition region counterparts one by one, and compute the Phillips multipliers to its regional regional monetary policy shock. In the left graph in Fig 4, each case are denoted with different combination of *N* and *ξ*. The benchmark Phillips multiplier curve, denoted as (*N*_*H*_, *ξ*_*H*_), is the red one. When we alter the firm measure from *N*_*H*_ into *N*_*L*_, the Phillips multiplier curve moves up a bit to the line with (*N*_*L*_, *ξ*_*H*_). In contrast, if the Calvo price parameter changes from *ξ*_*H*_ into *ξ*_*L*_, the Phillips multiplier curve jumps down to the line with (*N*_*H*_, *ξ*_*L*_).

Similarly, we can change the parameters *N*_*L*_ and *ξ*_*L*_ of low competition region into their high competition region counterparts one by one, and compute the Phillips multipliers to the low competition region monetary policy shock again. The results are displayed in the right graph of Fig 4. As in the case of high competition region, when the price adjustment parameter *ξ*_*L*_ changes into *ξ*_*H*_, the Phillips multipliers rise significantly.

Evidently, the shift of the Phillips multiplier is much larger when the nominal rigidity changes for both regions. Therefore, regional differences in the output and inflation trade-off are mainly driven by the nominal rigidity difference, and the sensitivity of trade-off to the real rigidity is small. Looking at the first points of the lines, we can conclude that the slope of Phillips curve is mainly determined by the nominal rigidity.

### 4.4 Robustness

In the previous analysis, we keep the price indexation same across regions. This allows us to conduct the counterfactual experiments on real rigidity *N* and price adjustment *ξ* easily and cleanly. In the following exercise, we relax this restriction and re-estimate the model allowing different regional price indexation.

**Fig 5:**
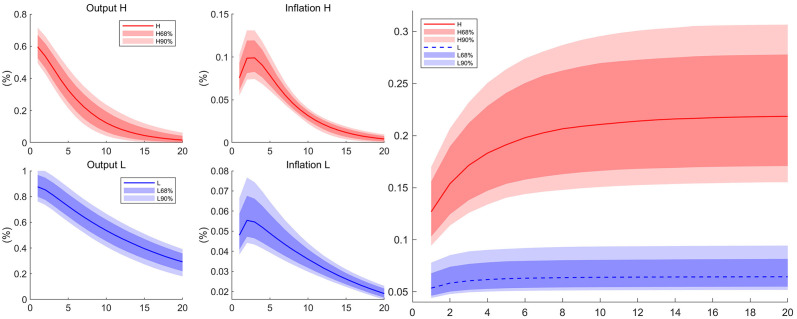
Impulse responses and Phillips multipliers to monetary policy shocks (*δ*_*d*_ relaxed). Estimated results of DSGE model with different regional price indexation *δ*_*d*_. The left four graphs depict the impulse responses to a positive one percent monetary policy shock. The right graph depicts the corresponding Phillips multipliers. The ratio immediately after the monetary policy shock is the slope of the Phillips curve. The solid lines are the median of the posterior distribution and the shaded areas are the corresponding confidence interval. *H* denotes the high-competition region and *L* the low-competition region.

Table 3 reports the estimated results after the price indexation is relaxed. The regional estimates of price indexation, denoted as *δ*_*j*,*d*_, is 0.53 for the high competition region and 0.21 for the low competition region, while it is 0.31 in the previous case, falling between these estimates. As for other parameters, relaxing the indexation does not change estimates too much from the previous case. In particular, the regional estimates of *ξ* are close to their counterparts in Table 2. Consequently, the estimated impulse responses to regional monetary policy shock and the Phillips multiplier shown in Fig 5 are close to benchmark results.

**Table 3:** Bayesian estimation results (allow *δ*_*d*_ be different among regions).

	Prior distribution			Posterior distribution			
	Distribution	Mean	Std.Dev.	Mean	Median	5%	95%
A. Nominal price ridigity							
*ξ*_*H*_	Beta	0.500	0.100	0.867	0.871	0.825	0.916
*ξ*_*L*_	Beta	0.500	0.100	0.949	0.952	0.938	0.962
*δ*_*H*,*d*_	Beta	0.400	0.100	0.525	0.528	0.379	0.687
*δ*_*L*,*d*_	Beta	0.400	0.100	0.207	0.203	0.115	0.289
B. Monetary policy							
*ϕ*_*H*,*π*_	Normal	-1.000	0.100	-1.193	-1.191	-1.319	-1.061
*ϕ*_*H*,*y*_	Normal	-0.300	0.100	-0.387	-0.386	-0.528	-0.256
*ϕ*_*H*,*m*_	Beta	0.400	0.100	0.449	0.451	0.353	0.554
*σ*_*H*,*m*_	Inv.Gamma	0.050	0.100	0.021	0.021	0.017	0.024
*ϕ*_*L*,*π*_	Normal	-1.000	0.100	-0.900	-0.901	-1.050	-0.756
*ϕ*_*L*,*y*_	Normal	-0.300	0.100	-0.379	-0.380	-0.514	-0.238
*ϕ*_*L*,*m*_	Beta	0.400	0.100	0.525	0.528	0.379	0.687
*σ*_*L*,*m*_	Inv.Gamma	0.050	0.100	0.049	0.049	0.038	0.059
C. Shocks							
*ρ*_*H*,*a*_	Beta	0.700	0.100	0.961	0.963	0.951	0.974
*ρ*_*H*,*ψ*_	Beta	0.500	0.100	0.289	0.288	0.184	0.379
*σ*_*H*,*a*_	Inv.Gamma	0.050	0.100	0.123	0.119	0.086	0.156
*σ*_*H*,*ψ*_	Inv.Gamma	0.050	0.100	0.216	0.201	0.094	0.311
*ρ*_*L*,*a*_	Beta	0.700	0.100	0.617	0.613	0.414	0.803
*ρ*_*L*,*ψ*_	Beta	0.500	0.100	0.498	0.497	0.358	0.639
*σ*_*L*,*a*_	Inv.Gamma	0.050	0.100	0.455	0.457	0.192	0.685
*σ*_*L*,*ψ*_	Inv.Gamma	0.050	0.100	0.040	0.034	0.013	0.060

The posterior distribution is constructed by the Metropolis-Hastings algorithm with a single chain of 100000 draws, after dropping 100000 draws as a burn-in.

**Fig 6:**
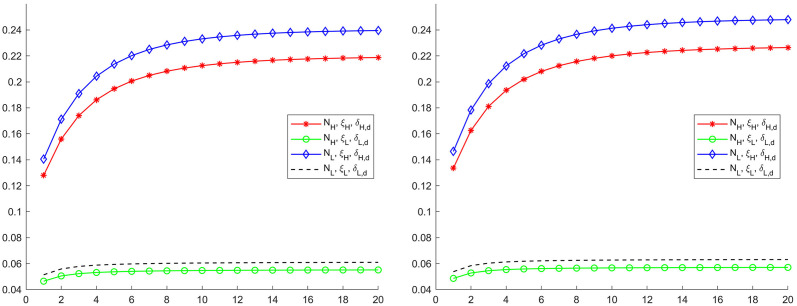
Phillips multipliers in counterfactual experiments (*δ*_*d*_ relaxed). Each graph displays four cases of Phillips multipliers after a positive regional money growth rate shock. The ratio immediately after the monetary policy shock is the slope of the Phillips curve. The line labeled with (*N*_*j*_, *ξ*_*j*_, *δ*_*j*_) denotes the Phillips multipliers computed under parameters from the high-competition (*j* = *H*) or the low-competition region (*j* = *L*). The left graph reports results for the high competition region and the right graph reports results for the low competition region.

We next conduct the counterfactual experiments for each regions. Different from previous benchmark case, we now have two parameters related with the nominal rigidity. The counterfactual experiments for high competition regions are displayed in the left graph of Fig 6, and the results corresponding to low competition regions are shown in the right graph. The line denoted with (*N*_*H*_, *ξ*_*H*_, *δ*_*H*,*d*_) is the Phillips multiplier for high competition region. It shifts upward a bit when we change the real rigidity from *N*_*H*_ into *N*_*L*_, while it moves downward a lot when we change the nominal rigidity from (*ξ*_*H*_, *δ*_*H*,*d*_) into (*ξ*_*L*_, *δ*_*L*,*d*_). When all three parameters are altered into the ones in low competition region, the line moves to the dashed one. Similar results are also found in the counterfactual experiments for the low competition region but in an opposite way. High level of competition raises real rigidity and lowers nominal rigidity, but it is the nominal rigidity that dominates the regional differences. Therefore, the high level of competition increase the slope of regional Phillips curve.

**Fig 7:**
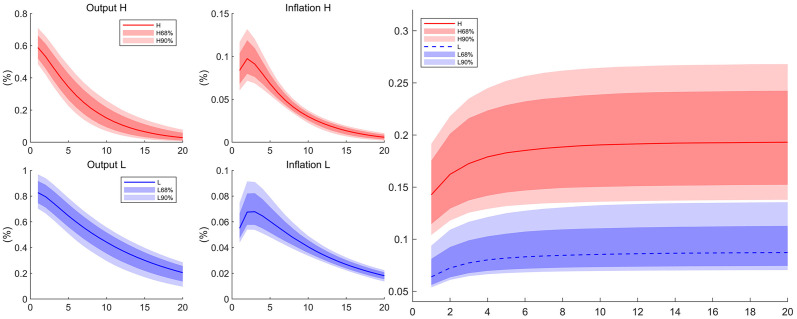
Impulse responses and Phillips multipliers to monetary policy shocks (*η* = 0). Estimated results of DSGE model with restriction *η* = 0. The left four graphs depict the impulse responses to a positive one percent monetary policy shock. The right graph depicts the corresponding Phillips multipliers. The ratio immediately after the monetary policy shock is the slope of the Phillips curve. The solid lines are the median of the posterior distribution and the shaded areas are the corresponding confidence interval. *H* denotes the high-competition region and *L* the low-competition region.

Unlike the Dixit-Stiglitz aggregator, the demand elasticity of each firm under the Kimball aggregator increases in its price relative to its competitors. To substantiate that our empirical findings are not merely a consequence of the unique characteristics of the Kimball aggregator, we set the parameter *η* to 0. This adjustment causes the model to degenerate to the standard constant elasticity of substitution (CES) aggregator framework. We then re-estimate the model under these conditions as a further robust check. Table 4 reports the estimated results with *η* = 0. By restricting *η* to 0, the model no longer separately captures the effect of competition on firms’ price setting through the real rigidity channel. In this case, estimates of the frequency of price adjustments are a reflection of the net effect of competition through nominal and real rigidity. Compared to the baseline results in Table 2, when *η* = 0, the estimation of nominial rigidity in *H* region *ξ*_*H*_ increases while *ξ*_*L*_ remains essentially unchanged. This outcome reflects the fact that the effect of real rigidity is more pronounced in high competition region. The parameter *δ*_*d*_ in Table 4 also exhibits a decrease, which corresponds to an increase in the estimate of nominal rigidity. Overall, the estimates of *ξ*_*H*_, *ξ*_*L*_ and *δ*_*d*_ demonstrate minimal variation, reinforcing the argument that nominal rigidity is the primary determinant of the slope of the regional Phillips curve. The responses to regional monetary policy shock and the Phillips multiplier in Fig 7 remain closely aligned with the benchmark results, suggesting that the findings of our paper are robust to the specification changes induced by setting *η* to 0.

**Table 4:** Bayesian estimation results (*η* = 0).

	Prior distribution			Posterior distribution			
	Distribution	Mean	Std.Dev.	Mean	Median	5%	95%
A. Nominal price ridigity							
*ξ*_*H*_	Beta	0.500	0.100	0.877	0.880	0.831	0.922
*ξ*_*L*_	Beta	0.500	0.100	0.938	0.943	0.924	0.953
*δ*_*d*_	Beta	0.400	0.100	0.306	0.301	0.178	0.420
B. Monetary policy							
*ϕ*_*H*,*π*_	Normal	-1.000	0.100	-1.206	-1.204	-1.347	-1.072
*ϕ*_*H*,*y*_	Normal	-0.300	0.100	-0.383	-0.384	-0.512	-0.249
*ϕ*_*H*,*m*_	Beta	0.400	0.100	0.441	0.442	0.340	0.541
*σ*_*H*,*m*_	Inv.Gamma	0.050	0.100	0.022	0.022	0.018	0.025
*ϕ*_*L*,*π*_	Normal	-1.000	0.100	-0.890	-0.888	-1.037	-0.737
*ϕ*_*L*,*y*_	Normal	-0.300	0.100	-0.384	-0.385	-0.526	-0.233
*ϕ*_*L*,*m*_	Beta	0.400	0.100	0.548	0.553	0.387	0.702
*σ*_*L*,*m*_	Inv.Gamma	0.050	0.100	0.051	0.050	0.039	0.061
C. Shocks							
*ρ*_*H*,*a*_	Beta	0.700	0.100	0.971	0.973	0.962	0.984
*ρ*_*H*,*ψ*_	Beta	0.500	0.100	0.302	0.301	0.198	0.398
*σ*_*H*,*a*_	Inv.Gamma	0.050	0.100	0.116	0.111	0.080	0.147
*σ*_*H*,*ψ*_	Inv.Gamma	0.050	0.100	0.246	0.226	0.109	0.351
*ρ*_*L*,*a*_	Beta	0.700	0.100	0.631	0.626	0.410	0.854
*ρ*_*L*,*ψ*_	Beta	0.500	0.100	0.497	0.497	0.354	0.640
*σ*_*L*,*a*_	Inv.Gamma	0.050	0.100	0.354	0.355	0.110	0.522
*σ*_*L*,*ψ*_	Inv.Gamma	0.050	0.100	0.046	0.037	0.014	0.070

The posterior distribution is constructed by the Metropolis-Hastings algorithm with a single chain of 100000 draws, after dropping 100000 draws as a burn-in.

## 5. Conclusion

Our paper examines the impacts of competition through real and nominal rigidity on the slope of the Phillips curve across regions in China. More intense market competition decreases nominal rigidity, steepening the Phillips curve, while it increases real rigidity, flattening the curve. Our Bayesian estimation of a New Keynesian model with Kimball aggregator and Calvo price-setting finds that the high competition region has a steeper Phillips curve. Through the counterfactual experiments, we find that it is the nominal rigidity that mainly determines the slope of the Phillips curve between the high and low competition regions. Real rigidity from competition only accounts for a small part of the difference in slopes between regions. The high level of competition intense the regional inflation-output trade-off and steeper the regional Phillips curve.

In addition to the degree of market competition, factors such as the degree of openness and labor mobility also affect the slope of the regional Phillips curve. A higher degree of regional openness can increase the susceptibility of local output and inflation to international market influences. This can weaken the link between domestic inflation and local economic fluctuations, resulting in a decrease in the slope of the regional Phillips curve [42, 43]. Similarly, increased labour mobility and migration can also lead to a flatter Phillips curve [44, 45]. However, our analysis shows that regions with higher levels of market competition are mainly concentrated in the eastern coastal provinces of China, where the degree of openness and labor mobility is relatively higher compared to regions with lower competition levels. Thus, our findings cannot be explained by differences in regional openness or labor mobility. Considering the current situation in China, one possible explanation for the observed phenomenon is the implementation of government price controls in the central and western regions. This may result in greater price rigidity and a flatter Phillips curve in these areas. However, the level of government price management is often linked to the degree of marketization and competitiveness of the local market. Stronger government control over prices tends to occur in areas with lower levels of marketization and weaker local market competition. This is consistent with our viewpoint to some extent.

In our current approach, competition does not endogenously affect the frequency of price adjustments, which simplifies our model and estimation. We proceed with the Calvo price-setting and let the data determine the frequency across regions in the structural estimation. We leave the extension of model with endogenous frequency of price adjustments to the degree of competition for the future work. We leave the extension of the model to endogenous price adjustment frequency for the future work.

This study shows that disparities in domestic market segmentation and the intensity of regional competition engender divergent responses in regional prices and outputs to identical monetary policy interventions. These findings have practical implications for policymakers. First, in formulating monetary policy, the central bank should take into account the differences between regions in terms of market competitiveness. Theoretically, the central bank should implement region-specific policy rates based on specific regional economic conditions and Phillips curve characteristics. However, this may be challenging to implement in practice. Second, it is necessary for the government to establish a unified domestic market and gradually address the current situation of market segmentation and differences in regional market competition. This can be achieved by removing regional market barriers and enabling the free movement of goods and factors of production.

Our paper also offers advice for firms in highly competitive markets. Before setting prices, companies can conduct comprehensive market research to gain insight into customer needs and competitor strategies. Accurate and detailed market information can assist companies in anticipating market changes and gaining a competitive advantage. Furthermore, firms can innovate to offer distinctive products that differentiate themselves from their competitors and reduce their substitutability in a fiercely competitive market.

## Supporting information

S1 Appendix Data source and model details. (PDF)

S1 Dataset Dataset. (XLSX)
