# Supplementary material for: Competition and regional Phillips curve: Evidence from China
Source: PLoS One. 2024 May 16;19(5):e0301546. doi: 10.1371/journal.pone.0301546 (PMC11098360; doi:10.1371/journal.pone.0301546)
Supplement: S1 Appendix — (PDF) [file pone.0301546.s001.pdf]

S1 Appendix

Data description

Our data contain quarterly time series of provincial GDP, provincial CPI, and national monetary indicators from China.The sample period starts from 2009Q1 to 2019Q4 to avoid the large shock of 2007 financial crisis and 2019 Covid pandemic.

The series of province-level gross regional product are collected from the National Bureau of Statistics of China (NBSC)(https://www.stats.gov.cn/).

The province-level month-on-month CPI is downloaded from the WIND database as the measurement for inflation. We don't use the GDP deflator or PPI because the former is only available at the national level, and the latter starts from 2015.

In our baseline estimation, we use the log difference of M2 as a measurement for money growth rate. The results remain robust when using aggregate financing as a measurement of the money supply. Monthly data on M2 are mainly derived from the Wind database. Official quarterly data on the Aggregate Financing: Stock have only been published since 2014. Therefore, as an alternative, we use the data constructed by Chang et al. [1], which includes quarterly data for Aggregate Financing: Stock since 2002 (https://www.atlantafed.org/cqer/research/china-macroeconomy).

For all the data in the estimation, we seasonally adjusted the series using the X-13 method and then remove the local mean via a bi-weight filter following Stock and Watson [2].

Data collected from Wind database are not in the public domain but are commercially available from Wind Information Co., Ltd.,(https://www.wind.com.cn/portal/en/EDB/index.html).

The data used in the Bayesian estimation are summarised in Table S1.

Table S1. Data and data sources

Variable	Data series	Data source
Output	Gross Regional Product	NBSC
Inflation	CPI,month on month	Wind
Monetary Policy	China Money Supply M2	Wind
Monetary Policy	Aggregate Financing	Chang et al.[1]

Groups of provinces

To examine how market competition affects regional Phillips curves, we divide the provinces in mainland China into two groups, the high competition region (H) and low competition region (L), according to the number of firms per inhabitants at the province level. Data on the number of provincial enterprises are derived from Chinese Annual Survey of Industrial Firms (ASIF), and we obtain information on population from the NBSC.

Data from Chinese Annual Survey of Industrial Firms are collected by the National Bureau of Statistics of China. These data are confidential and can only be accessed by permission of China's National Bureau of Statistics (https://www.stats.gov.cn/) or their authorized data center (https://www.ccerdata.cn/special).

The high competition region group includes: Beijing, Tianjin, Hebei, Liaoning, Jilin, Shanghai, Jiangsu, Zhejiang, Fujian, Shandong, Guangdong, Guangxi, Hainan, Chongqing, Xinjiang.

The low competition region group includes: Shanxi, Inner Mongolia, Heilongjiang, Anhui, Jiangxi, Henan, Hubei, Hunan, Sichuan, Guizhou, Yunnan, Shaanxi, Gansu, Qinghai, Ningxia.

Hong Kong, Macau and Tibet are excluded because their economy composition is very different from others.

Derivation of the New Keynesian Phillips Curve

For simplicity, we ignore the subscript j, which represents region identification.

When firm i can re-optimize its price in period t, it maximizes the expected discounted value of profit,

$$E_t \sum_{k=0}^{\infty} \{\xi^k [Q_{t+k} I_{t,t+k} P_{i,t} Y_{t+k} x_{i,t+k} - S(Y_{t+k} x_{i,t+k})]\} \tag{S1}$$

taking the process of stochastic discount factor $Q_{t+k} = \beta^k (\frac{C_{t+k}}{C_k})^{-\sigma} \frac{P_t}{P_{t+k}}$, the market demand $x_{i,t+k} = \frac{1}{1+\eta} (\frac{I_{t,t+k} P_{i,t}}{P^c_{t+k}})^{\frac{1}{\gamma_t - 1}} + \frac{\eta}{1+\eta}$, the aggregate prices, the wage rate, and price indexation $I_{t,t+k} = \prod_{s=0}^{k-1} (1 + \Pi_{t+s})^{\delta_D}$ as given. $S(\cdot)$ denotes the firm's cost function.

The first-order condition for the firm's price is

$$\begin{aligned} 0 = \quad & \frac{1}{P^o_{i,t}} E_t \textstyle\sum_{k=0}^{\infty} \{\xi^k Q_{t+k} Y_{t+k} x_{i,t,t+k})(\theta_{i,t+k} - 1) * \\ & [\frac{I_{t,t+k} P^o_{i,t}}{P_{t+k}} - \mu_{i,t+k} MCR_{i,t+k} (Y_{t+k} x_{i,t+k})]\}, \end{aligned} \tag{S2}$$

where $P^o_{i,t}$ is the optimal price chosen by firm i at time t, and MCR_t is the real marginal cost at time t. $\theta_i(\frac{I_{t,t+k} P^o_{i,t}}{P_{t+k}}) = -x'(\frac{I_{t,t+k} P^o_{i,t}}{P_{t+k}}) * (\frac{I_{t,t+k} P^o_{i,t}}{P_{t+k}}) / x(\frac{I_{t,t+k} P^o_{i,t}}{P_{t+k}})$ and $\mu_i(\frac{I_{t,t+k} P^o_{i,t}}{P_{t+k}})) = \theta_i(\frac{I_{t,t+k} P^o_{i,t}}{P_{t+k}}) / (\theta_i(\frac{I_{t,t+k} P^o_{i,t}}{P_{t+k}}) - 1)$.

log-linearized Eq S2 around zero inflation steady state,

$$\begin{aligned} 0 = \quad & E_t \textstyle\sum_{k=0}^{\infty} \{(\xi\beta)^k [(\hat{rp}^o_{i,t} + \delta_D(\pi_t + ... + \pi_{t+k-1}) - (\pi_{t+1} + ... + \pi_{t+k})) \\ & -(\hat{\mu}_{i,t,t+k} + \hat{mcr}_{i,t+k})]\} \end{aligned} \tag{S3}$$

The variables with hat represent their logarithmic deviation from their steady state. where $\hat{rp}^o_{i,t} = log(\frac{P^o_{i,t}}{P_t}) - log(\frac{P_i}{P})$.

Extending the terms in the above equation:

$$\begin{aligned} \hat{x}_{t,t+k} = \quad & s\frac{1}{\gamma-1}(\hat{rp}^o_t + \delta_D(\pi_t + ... + \pi_{t+k-1}) - (\pi_{t+1} + ... + \pi_{t+k}) \\ & + \pi_{t+k} - \pi^c_{t+k} + \frac{1}{\gamma-1} log(N) \hat{\psi}_{t+k}) \end{aligned} \tag{S4}$$

,where$s = \frac{(P_i/P^c)^{\frac{1}{\gamma-1}}}{(P_i/P^c)^{\frac{1}{\gamma-1}} + \eta} = \frac{(N)^{-\frac{1}{\gamma}}}{(N)^{-\frac{1}{\gamma}} + \eta}$.

π_t and π^c_t are first order approximations around the steady state.

According to the price adjustment rule and the definition of the price index:

$$\hat{rp}^o_{i,t} \quad = \frac{\xi}{1-\xi}(\pi_t - \delta_D \pi_{t-1}) \tag{S5}$$

Log-linearizing μ_{t+k} around the steady state:

$$\hat{\mu}_{t,t+k} = (-\frac{1}{\bar{\theta}-1})\hat{\theta}_{t,t+k} = e_{\mu}^{\gamma}\hat{\psi}_{t+k} + e_{\mu}^{x}\hat{x}_{t,t+k}, \tag{S6}$$

where e_{μ}^{γ} is the elasticity of the markup to γ at the steady state, and e_{μ}^{x} is the elasticity of the markup to firm's market share at the steady state.

$$\hat{mcr}_{i,t+k} = \hat{mcr}_{t+k} + e_{mc}^{y}(\hat{x}_{t,t+k}) \tag{S7}$$

where e_{mc}^{y} is the elasticity of the real marginal cost to firm's own output at the steady state.

$$\hat{mcr}_{t+k} = (\sigma + \frac{\phi+\alpha}{1-\alpha})\tilde{y}_t \tag{S8}$$

$\tilde{y}_t = \hat{y}_{t+k} - \hat{y}_{t+k}^{n}$ is the log-deviation of output from its natural level.

Substitute Eq S5 $\sim$ S8 into Eq S3 and after rearrange, we can get the New Keynesian Phillips Curve:

$$\pi_t = \frac{\delta_d}{1+\delta_d\beta}\pi_{t-1} + \frac{\beta}{1+\delta_d\beta}E_t\pi_{t+1} + \Theta\tilde{y}_t + k_j\hat{\psi}_t \tag{S9}$$

where $\Theta = \frac{(1-\xi\beta)(1-\xi)}{\xi(1+\delta_d\beta)}\frac{\sigma+\frac{\varphi+\alpha}{1-\alpha}}{1+\bar{\theta}(e_{\mu}^{x}+e_{mc}^{y})}$ and $k = \frac{(1-\xi\beta)(1-\xi)}{\xi(1+\delta_d\beta)}\frac{e_{\mu}^{\gamma}-e_{mc}^{\gamma}}{1+\bar{\theta}(e_{\mu}^{x}+e_{mc}^{y})}$.

Equilibrium conditions

To simplify our derivation and consistent with our calibration, we set $\sigma = \nu$ in our model. The log-linearized equations of the model are as follows,

$$\hat{c}_{j,t} = E_t\hat{c}_{j,t+1} - \tfrac{1}{\sigma}E_t(\hat{i}_{j,t} - \hat{\pi}_{j,t+1}) \tag{S10}$$

$$\varphi\hat{l}_{j,t} = \hat{rw}_{j,t} - \sigma\hat{c}_{j,t} \tag{S11}$$

$$\hat{gm}_{j,t} = \pi_{j,t} + \hat{c}_{j,t} - \hat{c}_{j,t-1} - \tfrac{1}{\nu(1+\bar{i})}(i_{j,t} - i_{j,t-1}) \tag{S12}$$

$$\hat{r}_{j,t}^{n} = \sigma E_t(\hat{y}_{j,t+1}^{n} - \hat{y}_{j,t}^{n}) \tag{S13}$$

$$\hat{y}_{j,t}^{n} = \tfrac{\varphi+1}{1+\varphi+\alpha(\sigma-1)}\hat{a}_{j,t} \tag{S14}$$

$$\hat{gy}_{j,t} = \hat{y}_{j,t} - \hat{y}_{j,t-1} \tag{S15}$$

$$\hat{y}_{j,t} = \hat{c}_{j,t} \tag{S16}$$

$$\hat{y}_{j,t} = \hat{a}_{j,t} + (1-\alpha)\hat{l}_{j,t} \tag{S17}$$

$$\hat{mcr}_{j,t} = \hat{rw}_{j,t} - (\hat{a}_{j,t} - \alpha\hat{l}_{j,t}) \tag{S18}$$

$$\pi_{j,t} = \tfrac{\delta_d}{1+\delta_d\beta}\pi_{j,t-1} + \tfrac{\beta}{1+\delta_d\beta}E_t\pi_{j,t+1} + \Theta_j\tilde{y}_{j,t} + k_j\hat{\psi}_{j,t} \tag{S19}$$

$$\tilde{y}_{j,t} = \hat{y}_{j,t} - \hat{y}_{j,t}^{n} \tag{S20}$$

$$\hat{\gamma}_{j,t} = -\hat{\psi}_{j,t} \tag{S21}$$

$$\hat{gm}_{j,t} = \phi_{j,m}\hat{gm}_{j,t-1} + (1-\phi_{j,m})(\phi_{j,\pi}\pi_{j,t} + \phi_{j,y}\hat{gy}_{j,t}) + \varepsilon_{j,t}^{m} \tag{S22}$$

$$\hat{gm}_{t} = \omega\hat{gm}_{H,t} + (1-\omega)\hat{gm}_{L,t} \tag{S23}$$

$$\hat{a}_{j,t} = \rho_{j,a}\hat{a}_{j,t-1} + \varepsilon_{j,t}^{a} \tag{S24}$$

$$\hat{\psi}_{j,t} = \rho_{j,\psi}\hat{\psi}_{j,t-1} + \varepsilon_{j,t}^{\psi} \tag{S25}$$

where $j \in \{H, L\}$, and the structural shocks are i.i.d, and normally distributed,

$$\varepsilon_{j,t}^{a} \sim N(0, \sigma_{j,a}^{2}) \tag{S26}$$

$$\varepsilon_{j,t}^{m} \sim N(0, \sigma_{j,m}^{2}) \tag{S27}$$

$$\varepsilon_{j,t}^{\psi} \sim N(0, \sigma_{j,\psi}^{2}) \tag{S28}$$

Additional results for the benchmark estimation

Figure S1 displays the prior and posterior distributions of the parameters in our benchmark estimation where we use the growth rate of M2 as measure of monetary policy and require the indexation parameters are same across regions.

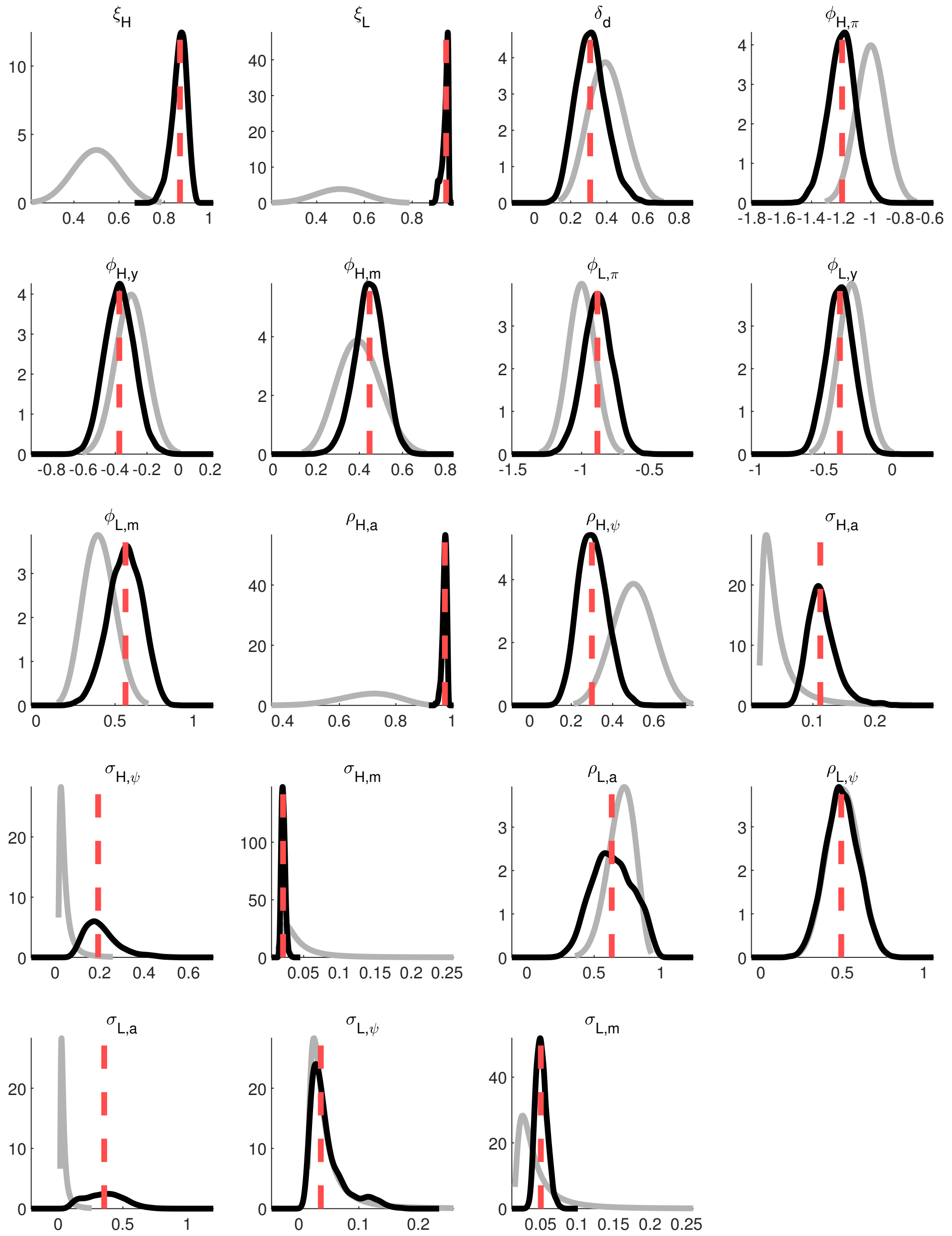

Fig S1. The prior and posterior distribution of parameters. The gray lines represent the prior distributions. The dark lines represent the posterior distributions. The vertical dash lines locate the medians of the posterior distributions.

Bayesian estimation results with Aggregate Financing

In the main text, we estimate the monetary policy rule with the growth rate of M2. To check the robust of our results, we re-estimate our baseline DSGE model with a different measurement of monetary policy supply. In this part, We use the Aggregate Financing as the measurement of monetary supply.

The Bayesian estimation results are reported in Table S2. All the parameters are almost the same as our benchmark estimation, especially the Calvo pricing parameters. The regional monetary policy parameters are very close to the previous case too. As a result, the IRFs to the regional monetary policy shock and the Phillips multipliers in Figure S2 are similar to those in the benchmark. Our results in the main text are robust to different measurements of monetary quantity.

Table S2. Bayesian Estimation results (Use Aggregate Financing as the measurement of Finance)

	Prior distribution			Posterior distribution			
	Distribution	Mean	Std.Dev.	Mean	Median	5%	95%
A. Nominal price ridigity							
ξ_H	Beta	0.500	0.100	0.874	0.878	0.832	0.921
ξ_L	Beta	0.500	0.100	0.946	0.949	0.931	0.967
δ_d	Beta	0.400	0.100	0.362	0.357	0.217	0.488
B. Monetary policy							
$\phi_{H,\pi}$	Normal	-1.000	0.100	-1.210	-1.209	-1.342	-1.068
$\phi_{H,y}$	Normal	-0.300	0.100	-0.351	-0.351	-0.490	-0.218
$\phi_{H,m}$	Beta	0.400	0.100	0.264	0.262	0.171	0.348
$\sigma_{H,m}$	Inv.Gamma	0.050	0.100	0.037	0.036	0.031	0.042
$\phi_{L,\pi}$	Normal	-1.000	0.100	-0.939	-0.936	-1.082	-0.791
$\phi_{L,y}$	Normal	-0.300	0.100	-0.358	-0.358	-0.506	-0.216
$\phi_{L,m}$	Beta	0.400	0.100	0.486	0.487	0.334	0.637
$\sigma_{L,m}$	Inv.Gamma	0.050	0.100	0.063	0.062	0.050	0.075
C. Shocks							
$\rho_{H,a}$	Beta	0.700	0.100	0.973	0.974	0.964	0.984
$\rho_{H,\psi}$	Beta	0.500	0.100	0.323	0.320	0.219	0.419
$\sigma_{H,a}$	Inv.Gamma	0.050	0.100	0.126	0.122	0.087	0.158
$\sigma_{H,\psi}$	Inv.Gamma	0.050	0.100	0.229	0.213	0.112	0.334
$\rho_{L,a}$	Beta	0.700	0.100	0.498	0.489	0.323	0.666
$\rho_{L,\psi}$	Beta	0.500	0.100	0.499	0.500	0.360	0.644
$\sigma_{L,a}$	Inv.Gamma	0.050	0.100	0.545	0.513	0.227	0.817
$\sigma_{L,\psi}$	Inv.Gamma	0.050	0.100	0.039	0.033	0.014	0.058

The posterior distribution is constructed by the Metropolis-Hastings algorithm with a single chain of 100000 draws, after dropping 100000 draws as a burn-in.

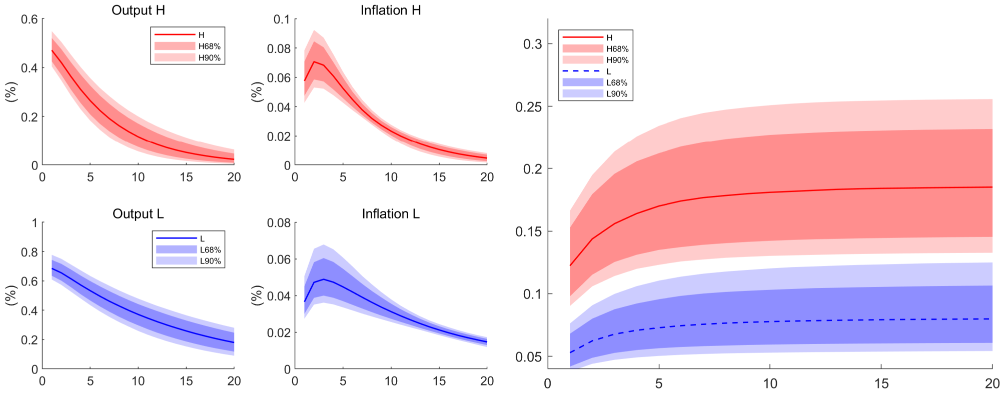

Fig S2. Impulse responses and Phillips multipliers to monetary policy shocks (Aggregate Financing). Estimated results of the DSGE model using Aggregate Financing as a measure of money supply. The left four graphs depict the impulse responses to a positive one percent monetary policy shock. The right graph depicts the corresponding Phillips multipliers. The ratio immediately after the monetary policy shock is the slope of the Phillips curve. The solid lines are the median of the posterior distribution and the shaded areas are the corresponding confidence interval. H denotes the high-competition region and L the low-competition region.

References

1. Chang, C., Chen, K., Waggoner, D. & Zha, T. Trends and cycles in China's macroeconomy. *NBER Macroeconomics Annual.* **30**, 1-84 (2016) https://doi.org/10.3386/w21244

2. Stock, J. & Watson, M. Disentangling the Channels of the 2007-2009 Recession. (National Bureau of Economic Research,2012) https://doi.org/10.3386/w18094
